# Management of belantamab mafodotin-associated corneal events in patients with relapsed or refractory multiple myeloma (RRMM)

**DOI:** 10.1038/s41408-021-00494-4

**Published:** 2021-05-26

**Authors:** Sagar Lonial, Ajay K. Nooka, Praneetha Thulasi, Ashraf Z. Badros, Bennie H. Jeng, Natalie S. Callander, Heather A. Potter, Douglas Sborov, Brian E. Zaugg, Rakesh Popat, Simona Degli Esposti, Julie Byrne, Joanna Opalinska, January Baron, Trisha Piontek, Ira Gupta, Reza Dana, Asim V. Farooq, Kathryn Colby, Andrzej Jakubowiak

**Affiliations:** 1 Emory University, Winship Cancer Institute, Atlanta, GA USA; 2 Emory Eye Center, Emory University, Atlanta, GA USA; 3 University of Maryland School of Medicine, Baltimore, MD USA; 4 Department of Ophthalmology and Visual Sciences, University of Maryland School of Medicine, Baltimore, MD USA; 5 University of Wisconsin, Carbone Cancer Center, Madison, WI USA; 6 University of Wisconsin, Madison, WI USA; 7 Huntsman Cancer Institute, University of Utah, Salt Lake City, UT USA; 8 Moran Eye Center, University of Utah, Salt Lake City, UT USA; 9 University College London Hospitals, NHS Foundation Trust, London, UK; 10 NIHR Biomedical Research Centre at Moorfields Eye Hospital NHS Foundation Trust and UCL Institute of Ophthalmology, London, UK; 11 GlaxoSmithKline, Upper Providence, PA USA; 12 Massachusetts Eye and Ear, Harvard Medical School, Boston, MA USA; 13 University of Chicago Medical Center, Chicago, IL USA; 14 New York University Grossman School of Medicine, New York, NY USA

**Keywords:** Cancer, Haematological cancer

## Abstract

Belantamab mafodotin (belamaf) demonstrated deep and durable responses in patients with heavily pretreated relapsed or refractory multiple myeloma (RRMM) in DREAMM-2 (NCT03525678). Corneal events, specifically keratopathy (including superficial punctate keratopathy and/or microcyst-like epithelial changes (MECs), eye examination findings with/without symptoms), were common, consistent with reports from other antibody–drug conjugates. Given the novel nature of corneal events in RRMM management, guidelines are required for their prompt identification and appropriate management. Eye examination findings from DREAMM-2 and insights from hematology/oncology investigators and ophthalmologists, including corneal specialists, were collated and used to develop corneal event management guidelines. The following recommendations were formulated: close collaboration among hematologist/oncologists and eye care professionals is needed, in part, to provide optimal care in relation to the belamaf benefit–risk profile. Patients receiving belamaf should undergo eye examinations before and during every treatment cycle and promptly upon worsening of symptoms. Severity of corneal events should be determined based on corneal examination findings and changes in best-corrected visual acuity. Treatment decisions, including dose modifications, should be based on the most severe finding present. These guidelines are recommended for the assessment and management of belamaf-associated ocular events to help mitigate ocular risk and enable patients to continue to experience a clinical benefit with belamaf.

## Introduction

In recent decades, significant advancements have been made in the management of multiple myeloma (MM), with several new treatments approved and novel classes of agents being investigated^1,2^. However, MM remains incurable and new and effective therapies are needed^2,3^. At present, patients with MM are treated with three major drug classes: immunomodulatory agents, proteasome inhibitors (PIs), and anti-CD38 monoclonal antibodies (mAbs)^2^. Treatment responses and survival outcomes diminish with subsequent relapses and the prognosis is poor for patients with relapsed or refractory MM (RRMM), particularly patients who become refractory to anti-CD38 mAbs (median overall survival (OS): 6–9 months)^1,2,4^.

B-cell maturation antigen (BCMA) is a receptor specifically expressed on the cell surface of late-stage B cells and plasma cells^3^. BCMA activation induces B-cell proliferation, differentiation, and survival^3^. Considering the selective expression of the BCMA receptor and its impact on late-stage B cells, BCMA represents an ideal therapeutic target for plasma cell malignancies^3^. BCMA-targeted therapies under clinical development include antibody–drug conjugates (ADCs), bispecific T-cell engagers, and chimeric antigen receptor T-cell therapies^3^.

Belantamab mafodotin (BLENREP; GSK2857916 (belamaf)) is a first-in-class ADC consisting of an anti-BCMA mAb conjugated to the microtubule inhibitor monomethyl auristatin F (MMAF)^5^. Belamaf eliminates MM cells by a multimodal mechanism of action, including apoptosis, and antibody-dependent cell-mediated anti-myeloma responses, accompanied by release of markers characteristic of immunogenic cell death^5,6^. In the pivotal, Phase II, DREAMM-2 study (NCT03525678), patients refractory to immunomodulatory agents and PIs, and refractory and/or intolerant to anti-CD38 mAbs, received single-agent belamaf at 2.5 or 3.4 mg/kg^7^. As of the 13-month follow-up, the median duration of response (DoR; 11.0 months) and OS (13.7 months) estimates for the patients receiving the 2.5-mg/kg dose compared favorably with previously reported outcomes in patients with prior exposure to anti-CD38 therapies treated with selinexor plus dexamethasone (median DoR: 4.4 months; median OS: 8.6 months)^8,9^.

**Fig. 1:**
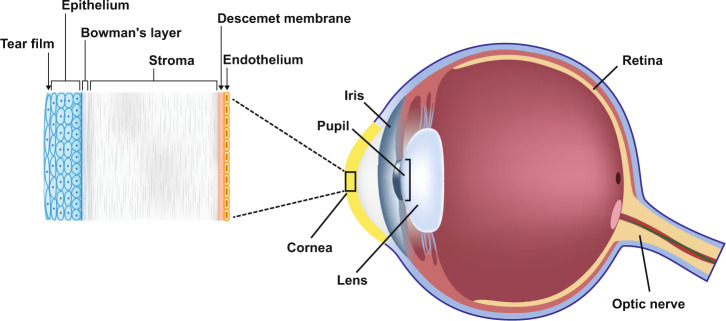
Anatomy of the eye, with focus on the cornea. AE adverse event, belamaf belantamab mafodotin. Belamaf-associated AEs were reported in the corneal epithelium, which is the outermost layer of the eye^7^.

Single-agent belamaf (2.5 mg/kg) had a manageable safety profile, with keratopathy (microcyst-like epithelial changes (MECs), changes in the corneal epithelium observed on eye examination with or without symptoms; 72%), thrombocytopenia (38%), and infusion-related reactions (21%) as commonly reported adverse events (AEs)^7,9^. The cornea is the transparent, anterior most structure of the eye and plays an important role in focusing light onto the retina (Fig. 1)^10^. In DREAMM-2, keratopathy (MECs) was typically described as superficial bilateral, microcyst-like lesions seen on slit lamp microscopy^11^. In some patients, MECs were first observed in the corneal periphery and progressed to the mid-periphery and subsequently the center^11^. The presence of keratopathy (MECs) in the corneal center tended to correlate with changes in vision, including subjective blurred vision^11^. Similar findings have been commonly described with other ADCs, particularly for MMAF-containing ADCs^11–13^. Keratopathy (MECs) observed with belamaf and other ADCs appears clinically distinct from other pathologies that are commonly encountered by corneal specialists^11^.

Keratopathy (MECs) observed on eye examination was frequent (72%; 68/95 patients) and occurred early in treatment (median time to onset: 37 days)^11^. These events often led to dose modifications (dose delays: 47%; dose reductions: 25%); however, only 1/95 (1%) patients receiving the 2.5-mg/kg dose discontinued treatment due to keratopathy (MECs), indicating that patients were able to remain on treatment while these events were monitored^11^. Clinical responses were maintained in over 80% of patients with prolonged dose delays (>63 days, equivalent to more than 3 cycles), suggesting that responses to belamaf are durable despite dose modifications^14^.

Based on the experience in DREAMM-2 and studies of other MMAF-containing ADCs, it is anticipated that patients will recover from corneal events^11^. In DREAMM-2, most patients (77%; 46/60) with a Grade ≥2 keratopathy (MEC) event recovered from their first event, with a median time to resolution of 86.5 days^11^. The majority of patients with Grade 3/4 events (84%; 37/44) either recovered or were recovering as of the last follow-up^14^. In patients with unrecovered Grade ≥2 events at last follow-up, 45% (14/31) are still receiving treatment or in follow-up, so monitoring for recovery is ongoing^14^. The remaining patients with unrecovered events (55%; 17/31) are no longer in follow-up due to death, study withdrawal, or loss to follow-up and therefore cannot be monitored for recovery^14^.

Corneal changes on eye examination were not always accompanied by patient symptoms or changes in best-corrected visual acuity (BCVA)^11^. Among all patients, 56% (53/95) had symptoms (e.g., blurred vision or dry eye symptoms) and/or a ≥2-line BCVA decline (in their better-seeing eye). Blurred vision and dry eye events were mainly grade 1/2. Seventeen patients (18%) experienced a clinically significant decline in BCVA to a Snellen score of 20/50 or worse in their better-seeing eye, at least once during or after the treatment period. In patients with normal/near normal vision at baseline, a Snellen score of 20/50 was used as a surrogate marker for a meaningful reduction in visual acuity. The majority (82%; 14/17) recovered as of the last follow-up. The median duration of these events was 21.5 days; therefore, most patients recovered after one assessment interval (conducted every 21 days during the trial). Of the remaining three patients with unrecovered events, one patient is receiving treatment and two patients are no longer in follow-up (one died due to disease progression; one withdrew from study). No patients treated with belamaf to date have had permanent vision changes or loss^15^.

Though ocular/corneal AEs are common with oncology therapeutics, keratopathy (MECs) is a novel treatment-emergent event that should be managed in patients with RRMM^11,16–20^. As described above, only a portion of patients with keratopathy (MECs) in DREAMM-2 experienced symptoms^11^. Given the high frequency and often asymptomatic nature of keratopathy (MECs) observed with belamaf treatment, close monitoring of corneal examination findings and changes in BCVA by an eye care professional is warranted. Therefore, it is important to provide hematologist/oncologists clear guidance on how to identify and monitor these events in patients receiving belamaf that they can use to guide treatment decisions. Here, we provide recommendations for hematologist/oncologists to actively manage keratopathy (MECs) based on clinical experience in DREAMM-2; reference should also be made to local labeling information for belamaf. A multidisciplinary team has been shown to improve care practices in the management of patients with hematological malignancies^21,22^. Therefore, we also propose recommendations to establish and facilitate a close collaboration between hematologist/oncologists and eye care professionals (ophthalmologists and optometrists) that will inform treatment decisions.

## Methods

### Keratopathy and visual acuity (KVA) scale development

Cumulative ocular/corneal safety data and related protocols from DREAMM-2 were reviewed by investigators (all hematologist/oncologists), eye care professionals who performed eye examinations of patients in DREAMM trials, and corneal specialists. Hematologist/oncologists and ophthalmologists also provided their expertise with managing RRMM treatment-related AEs and corneal surface diseases, respectively. The feedback from these experts was synthesized into a novel set of guidelines specific for belamaf-associated corneal events, called the KVA scale (Table 1).

**Table 1:** Recommended belamaf dose modifications based on eye examination findings per the KVA scale per the US prescribing information and the EU summary of product characteristics combined^22,23^.

Severity^a^	Corneal examination findings^b^			Change in BCVA due to treatment-related corneal findings	Recommended dose modifications
	Description	Presentation of MECs (based on density and location)	Example schematics of MECs by severity		
Grade 1/Mild	Mild superficial keratopathy^c^ (documented worsening from baseline) with or without symptoms	Mild density: non-confluent Location: predominantly (≥80%) peripheral		Decline from baseline of 1 line on Snellen Visual Acuity	Continue treatment at current dose
Grade 2/Moderate	Moderate superficial keratopathy^c^ with or without patchy microcyst-like deposits, sub-epithelial haze (peripheral), or a new peripheral stromal opacity	Moderate density: semi-confluent Location: predominantly (≥80%) paracentral		Decline from baseline by 2 or 3 lines (and Snellen Visual Acuity not worse than 20/200)	Withhold treatment until improvement in either exam findings or BCVA to Grade 1/mild • Resume at a reduced dose of 1.9 mg/kg
Grade 3/Severe	Severe superficial keratopathy^c^ with or without diffuse microcyst-like deposits involving the central cornea, sub-epithelial haze (central), or a new central stromal opacity	Severe density: confluent Location: predominantly (≥80%) central		Decline from baseline of more than 3 lines (and Snellen Visual Acuity not worse than 20/200)	Withhold treatment until improvement in either exam findings or BCVA to Grade 1/mild • Resume at a reduced dose of 1.9 mg/kg^d^
Grade 4/Severe	Corneal epithelial defect, including corneal ulcers. These should be managed promptly and as clinically indicated by an eye care professional	Not applicable, these events are not graded based on MECs		Snellen Visual Acuity worse than 20/200	Withhold treatment until improvement in examination findings and BCVA to Grade 1/mild. Consider treatment discontinuation based on a benefit–risk assessment. For worsening symptoms that are unresponsive to appropriate management, consider discontinuation • If continuing treatment, resume at a reduced dose of 1.9 mg/kg^d^

*BCVA* best-corrected visual acuity, *belamaf* belantamab mafodotin, *KVA* keratopathy and visual acuity, *MEC* microcystic-like epithelial changes.

^a^The severity category is defined by the more severely affected eye as both eyes may not be affected to the same degree. Prescribing physicians should refer to the guidelines for corneal adverse event management in their local labeling.

^b^The worst severity for MEC density or location should be used in grading. Grading is based on the worst finding in the worse-affected eye. These evaluations and examples do not apply to, or include, superficial punctate keratopathy.

^c^Patients may have superficial punctate keratopathy, MECs, or both. Keratopathy refers to superficial punctate keratopathy (revealed by fluorescein staining) or MECs (which may not stain with fluorescein). Fluorescein staining should be part of each eye examination, including the baseline examination. The worst grade for the keratopathy and the change in BCVA should be used to determine the grade of the corneal adverse event.

^d^The restarting dose for Grade 3/4 events per the US prescribing information.

### DREAMM-2 study design

DREAMM-2 is an ongoing, open-label, two-arm, Phase II study being conducted at 58 MM specialty centers in eight countries^7^. Full methodological details of DREAMM-2 were previously reported^7^. In brief, eligible patients with RRMM were randomized (1:1) to receive belamaf (BLENREP) 2.5 or 3.4 mg/kg every 3 weeks by intravenous infusion over 30 min or longer, on day 1 of each cycle. Patients received treatment until disease progression or unacceptable toxicity occurred.

Full inclusion/exclusion criteria were previously reported^7^. Eligible patients had RRMM disease progression after >3 prior lines of anti-myeloma treatment; and were refractory to both an immunomodulatory agent and a PI, and refractory and/or intolerant to an anti-CD38 mAb. Patients were excluded if they had corneal epithelial disease at screening (other than mild dry eye).

Eye examinations were conducted by an eye care professional at baseline and every 3 weeks during the study^7^. Eye examinations included, at minimum, an assessment of the cornea using a slit lamp and measurement of BCVA. Eye examination findings and changes in BCVA were graded based on the most severe finding per KVA scale. Ocular symptoms (e.g., blurred vision and dry eye symptoms) were collected by the hematologist/oncologist as part of the ongoing safety monitoring on treatment and in follow-up and graded using Common Terminology Criteria for Adverse Events version 4.03 (CTCAE v4.03). Eye examination findings and changes in BCVA were also graded per CTCAE v4.03. Dose modifications (dose delays and reductions) were based on the severity of these events per the KVA scale.

To potentially mitigate corneal events in DREAMM-2, patients were instructed to self-administer prophylactic corticosteroid eye drops (four times daily, starting 1 day pre-dose for a total of 7 days) and preservative-free lubricant eye drops (4–8 times daily during the study) in both eyes^7^. Throughout the study, patients were prohibited from using contact lenses.

DREAMM-2 was performed in accordance with the Declaration of Helsinki and Good Clinical Practice guidelines following approval by ethics committees and institutional review boards at each study site^7^. All patients provided written informed consent before enrollment.

## Results

### KVA scale development

Given the association of ocular events with MMAF-containing ADCs, including belamaf, a comprehensive approach was undertaken in DREAMM-2 to ensure the prompt detection and management of belamaf-associated corneal events^7,11–13^. In reviewing the procedures and corneal event safety data from DREAMM-2, along with their expertise gained on the study, trial investigators and eye care professionals, including corneal experts, sought to streamline and further refine the guidance for hematologist/oncologists and eye care professionals who will be caring for patients receiving belamaf. Trial investigators expressed that the guidance provided in the DREAMM-2 protocol for the grading and subsequent management of corneal events based on this grading was difficult to follow. Hematologist/oncologists are generally unfamiliar with ocular AE terminology and the slit lamp examination and would have difficulty accurately grading events without the assistance of eye care professionals. Direction was taken from eye care professionals who assessed patients in DREAMM-2, as well as corneal experts, who advised on simplification of the scale used in DREAMM-2, to allow for more uniform grading by eye care professionals.

### KVA scale: recommendations for identifying corneal events

Patients should undergo an eye examination at baseline, within 3 weeks before the first dose of belamaf^23,24^. Recommendations for follow-up eye examinations differ regionally due to requirements of the relevant regulatory bodies. In the USA, eye examinations must be conducted before every dose, whereas in the EU, eye examinations are only required before the first three treatment cycles. In both the USA and EU, additional eye examinations are required promptly as clinically indicated (e.g., on worsening of ocular symptoms). As belamaf is administered every 3 weeks, follow-up examinations can occur at least 1 week after the previous dose and within 2 weeks before the next dose (ideally as close to the next dose as possible). This recommended timing allows patients some flexibility in obtaining an appointment with an eye care professional and for the outcomes of that examination to be sent back to the treating hematologist/oncologist. We recommend that eye examinations should continue every 3 weeks during dose delays, whether the delay is due to a corneal event or any other non-ocular event.

Eye examinations for patients receiving belamaf should include both slit lamp examination of the cornea and BCVA assessment^23,24^. The slit lamp microscope allows detailed eye examination^25^. All slit lamp examinations should include fluorescein staining to show abnormalities in the corneal surface^25^. Patients who received belamaf may present with superficial punctate keratopathy, MECs, or both. Keratopathy refers to superficial punctate keratopathy (revealed by fluorescein staining), which is a broad term referring to non-inflammatory changes in the outer layer of the cornea. MECs are microscopic deposits that resemble cysts, which may not stain with fluorescein. Pupil dilation, to better assess the health of the retina and optic nerve, is required at baseline, but not at follow-up examinations, unless clinically indicated^26^. Dilation may lead to light sensitivity for a few hours after the eye examination^26^. Therefore, patients should be advised to bring sunglasses to their baseline examination and arrange for travel assistance after the examination. We have previously published guidance for eye care professionals on the appearance of keratopathy (MEC), using slit lamp microscopy^11^.

BCVA is the clarity/sharpness of vision a patient can achieve with correction measured using a Snellen chart^27^. Determining BCVA necessitates refraction, a test that measures the strength of the corrective lens needed to achieve precise focus^28^. Normal vision is considered to be a visual acuity score of 20/20 (if using feet) or 6/6 (if using meters)^27,29^. This means that at 20 feet or 6 meters from the chart, the patient can see what the average, healthy individual can see from that position^29^. For example, a patient with BCVA of 20/50 or 6/15 can see at 20 feet/6 meters what the average individual can see at 50 feet/15 meters away. The smallest line read correctly represents the patient’s BCVA^27,29^.

### KVA scale: recommendations for the grading of corneal events

To date, a linear relationship has not been observed between the severity of keratopathy (MECs) and changes in BCVA in patients receiving belamaf. Therefore, corneal events should be graded using the KVA scale, based on the worst finding of either keratopathy (MECs) seen on eye examination or BCVA assessment (Table 1)^23,24^. Here, we provide a combined summary of these grades (as used in the USA label/levels of severity are shown in the EU label) and dose modification guidelines in the current US and EU labels. Prescribing physicians should also refer to the guidelines for corneal AE management in their local labeling.

The severity of MECs is characterized by their location as well as density. MECs can start in the periphery of the cornea and in some cases migrate centrally^11^. Based on clinical observation, central changes to the cornea are more likely to be symptomatic and interfere with the patient’s vision. Grade 1/mild corneal events are characterized by the appearance of only a few, if any, MECs with a low density (non-confluent), and predominantly (≥80%) located in the periphery of the cornea. Grade 2/moderate MECs are moderately dense (semi-confluent) and predominantly located in the paracentral region of the cornea. Grade 3/severe MECs have a high density (confluent) and are predominantly located in the center of the cornea.

The worst severity for MEC density or location should be used for grading (Table 1). For example, the observation of semi-confluent (Grade 2/moderate) MECs, predominantly located in the center of the cornea (Grade 3/severe), would lead to this being graded as a Grade 3/severe corneal event. Grading is also based on the worst finding in the worse-affected eye, since both eyes may not be affected equally. For example, a patient with Grade 1/mild MECs in their left eye and Grade 2/moderate MECs in their right eye should be managed according to the guidelines for Grade 2/moderate MECs.

Using the KVA scale, Grade 2/moderate and Grade 3/severe corneal events can also include examination findings of sub-epithelial haze (cloudy appearance in the layer immediately below the corneal epithelium) or stromal opacity (cloudy appearance of the stroma, the middle layer of the cornea). Similar to MECs, central sub-epithelial haze or stromal opacity is more severe (i.e., Grade 3/severe) than peripheral events (i.e., Grade 2/moderate).

The change in BCVA should also be used to determine the KVA scale grade of the corneal event (Table 1). A 1-line worsening from baseline in BCVA on the Snellen chart represents a Grade 1/mild corneal event. Grade 2 (moderate) is a decline in BCVA of 2 or 3 lines from baseline, while Grade 3 (severe) is represented by a >3 line decline on the Snellen chart with BCVA not worse than 20/200 (a Grade 4 event is defined as BCVA worse than 20/200 (6/60))^23,24^.

A corneal epithelial defect, defined as loss of corneal epithelium, may result in significant ocular discomfort, visual impairment, and an increased risk of infection^30^. Corneal epithelial defects (as well as more severe events such as corneal ulceration (a defect accompanied by an infiltrate or significant haze) and corneal perforation) are considered Grade 4/severe events^18,23,24^. A BCVA worse than 20/200 (6/60) is also considered the most severe visual event and denotes a Grade 4/severe event^23,31^. In DREAMM-2, one patient in the 2.5 g/kg dose group and two patients in the 3.4 mg/kg dose group experienced a worsening of BCVA to ≥20/200 in their better-seeing eye^7^. All these events recovered to baseline. To date, there has been no permanent loss of vision in patients receiving belamaf.

### KVA scale: recommendations for management of corneal events

Belamaf corneal events are managed using dose modifications based on the KVA scale (Table 1)^23,24^. Treatment should be continued at the current dose for Grade 1/mild events without interruption. For Grade 2/moderate events, treatment should be delayed until the event improves to a Grade 1/mild event or resolves; treatment can be then resumed at a lower dose (1.9 mg/kg). Treatment should also be delayed for Grade 3/4 (severe) events until these improve to a Grade 1/mild event; treatment should be restarted at 1.9 mg/kg following improvement of a Grade 3/4 (severe) event; therapy can be reinitiated immediately after improvement to a Grade 1/mild event or after complete resolution.

For Grade 4/severe events, a benefit–risk assessment should be conducted to determine if permanent treatment discontinuation is required. If it is decided that treatment should be resumed following recovery of a Grade 4/severe event to Grade 1 or better, a reduced belamaf dose of 1.9 mg/kg is recommended. Treatment discontinuation should be considered for worsening symptoms that are unresponsive to appropriate management. For patients who have more than 1 dose delay/interruption due to corneal events, the belamaf dosing schedule of every 3 weeks should be maintained once events have improved and treatment restarted (i.e., the dosing schedule should not be modified in these patients).

In DREAMM-2, patients with a history of dry eye were more likely to develop keratopathy (MECs) compared with patients who did not have a history of dry eye^11^. Dry eye disease can manifest as punctate keratopathy and result in damage to the ocular surface^32^. However, the underlying etiology of belamaf-associated keratopathy (MECs) is not yet known^11^. Therefore, it is recommended that patients use preservative-free lubricant eye drops at least four times a day, starting before the first infusion and continuing until the end of treatment (Table 2)^23,24,33^. Additional supportive care measures may be considered, as recommended by the eye care professional^24^. An eye care professional may advise the patient to use bandage contact lenses, which are soft therapeutic contact lenses designed to serve as a protective barrier for the ocular surface^30^.

**Table 2:** Recommended management strategies for corneal events observed in patients receiving belamaf.

Strategy	Proposed purpose	Directions
Dose modifications (delays and reductions)	To limit the corneal exposure of belamaf, as the corneal surface regenerates and repairs itself^11^	• The eye care professional will determine the highest grade/severity of the corneal event per KVA scale (Table 1), clearly communicate this to the hematologist/oncologist, and continue to monitor these events^22,23^. The hematologist/oncologist will delay and/or reduce belamaf dose based on the KVA scale guidelines for the most severe eye examination finding
Regular use of preservative-free lubricant eye drops	To lubricate the cornea and relieve discomfort of subjective dry eye symptoms^29^; may decrease risk of corneal symptoms^23^	• Advise patients to use preservative-free lubricant eye drops at least 4 times a day in both eyes, starting with the first infusion and continuing until end of treatment^22,23^
Avoiding use of contact lenses unless clinically warranted. An eye care professional may direct the patient to use bandage contact lenses	Contact lenses may irritate the cornea^30^. Bandage contact lenses help protect and aide in repair of the corneal epithelium^27^	• Begin at the first infusion and continue throughout treatment^22,23^ • Relevant to both eyes

*Belamaf* belantamab mafodotin, *KVA* keratopathy and visual acuity.

Corticosteroid eye drops are not recommended as a management strategy, because these were shown to be ineffective in preventing keratopathy (MECs) in DREAMM-2^7^. Prophylactic topical corticosteroid eye drops have been used in studies with other ADCs to mitigate corneal events^11^. However, results have been mixed, and therefore no clear benefit has been demonstrated with this strategy to date.

### Multidisciplinary approach to manage corneal events

**Fig. 2:**
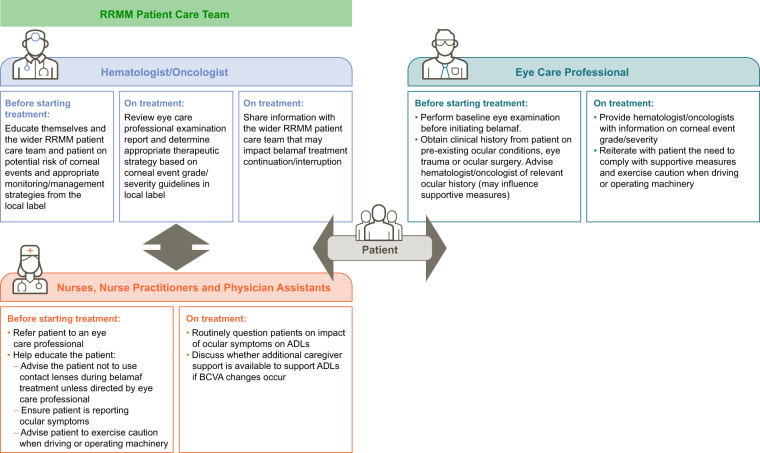
Flow chart of multidisciplinary approach to mananging corneal events with belamaf: health care professional roles.

In light of the known corneal events associated with single-agent belamaf for patients with RRMM, effective management requires close collaboration and clear communication between hematologist/oncologists, the wider RRMM patient care team (e.g., oncology nurses, nurse practitioners, and physician assistants), and eye care professionals to identify and manage these events. Figure 2 summarizes the roles of these professionals within a multidisciplinary approach to managing corneal events.

### The roles of the hematologist/oncologist and the RRMM patient care team

It is important that the RRMM care team managing a patient treated with belamaf is knowledgeable of the potential risks of corneal events. When the hematologist/oncologist decides to prescribe belamaf for their patient, they should educate themselves, the broader RRMM patient care team, and the patient on the potential risks of corneal events and how to monitor and appropriately manage these events. Educational materials for the RRMM patient care team and the patient are available through the Risk Evaluation and Mitigation Strategy program in the USA and the Risk Management Plan program in the EU^34,35^.

Specifically, the RRMM patient care team should advise the patient to not wear contact lenses during belamaf treatment, unless they are directed to by an eye care professional, as contact lens use can contribute to dry eye and other ocular complications^23,24,36^. The patient care team should advise patients to exercise caution when driving or operating machinery, since changes in visual acuity may occur^23,24^. Importantly, the team should discuss with the patient whether additional caregiver support is needed to maintain activities of daily living (ADLs; e.g., transportation to medical appointments) in the event of a transient change in visual acuity.

Given that corneal examination findings were not always accompanied by symptoms, the RRMM patient care team must help ensure adherence to the eye examination requirements at baseline and during treatment to accurately monitor for corneal changes^11,23,24^. The patient care team should also reiterate to the patient the importance of reporting ocular symptoms—in particular, blurred vision, subjective symptoms of dry eye, and changes in visual acuity. They should also routinely ask the patient about the impact of any ocular symptoms on ADLs, such as subjective blurred vision that interferes with reading or leads to difficulty driving^23,24^. Table 3 provides examples of questions for the team to use to assess the impact of ocular-related symptoms on a patient’s ADLs.

**Table 3:** Example questions to ask patients to facilitate reporting of new corneal-related AEs with belamaf treatment.

During conversations with patients regarding the effects of their treatment, it may be helpful to ask the following questions regarding new corneal AEs they may be experiencing with belamaf:
• Are you finding it difficult to read during the day due to your eyesight? Or at night?
• Have you noticed any problems with your eyesight while driving?
• Do you have any problems with your eyes or vision when using a computer/tablet/phone or watching the television?
◦ Have you needed to increase the font size on your devices so that you can see the text better?
• Have you noticed any vision changes or other symptoms when you engage in any other activities that are important to you?
• Have you experienced any pain or discomfort in or around your eyes?
• Are your eyes more sensitive than usual to light?
◦ Have you needed to turn off the lights or wear sunglasses indoors because you were more sensitive to light?
• Have you noticed any other symptoms related to your eyes or eyesight?
◦ Foreign body sensation?
◦ Watering eyes?
◦ Other (patient to indicate)?

*AE* adverse event.

The hematologist/oncologist should review the patient’s eye examination before dosing and determine the appropriate therapeutic strategy based on the grade of the most severe finding per the KVA scale (Table 1)^23,24^. Therefore, timely information exchange with eye care professionals is crucial for ensuring seamless treatment continuation, or delay if required to manage corneal events.

### The role of the eye care professional

The RRMM patient care team will refer the patient to an eye care professional to perform a baseline eye examination before starting belamaf treatment^23,24^. At the baseline examination, the eye care professional should ask about preexisting ocular conditions of interest, such as a history of glaucoma, cataract, any ocular surgeries (including laser-assisted in situ keratomileusis (LASIK) or refractive surgery) or eye trauma, diabetic retinopathy, and macular degeneration that might affect the BCVA. The eye care professional should then advise the hematologist/oncologist of any ocular history that would necessitate changes to supportive measures. For example, patients with a history of dry eye may be advised to use preservative-free lubricant eye drops more frequently than specified in the belamaf local label.

After follow-up examinations, the eye care professional should provide the hematologist/oncologist with the overall corneal event grade/severity using the KVA scale in their local label (Table 1)^23,24^. This overall score will represent the worst finding on either corneal examination or BCVA change assessment. The hematologist/oncologist will use this overall grade/severity to determine any appropriate dose modification; thus, it is important to clearly communicate these findings in terminology consistent with the local label.

If clinically warranted, the eye care professional may propose additional mitigation strategies for corneal events (e.g., bandage contact lenses or punctal plugs (to block tear duct drainage)). Eye care professionals should reiterate to patients the need to adhere to supportive care measures (e.g., preservative-free lubricant eye drops) and to exercise caution when driving or operating machinery^23,24^.

## Discussion

Belamaf is a first-in-class, anti-BCMA therapy that demonstrated deep and durable clinical responses as a single agent in patients with heavily pretreated RRMM in DREAMM-2^7^. Keratopathy (MECs), an eye examination finding with or without ocular symptoms, was the most common AE. The risk of keratopathy (MECs) does not appear to decrease over time and these events will recur with repeated dosing, so it is imperative to gain a better understanding of how to optimize corneal event management. Overall, corneal events were manageable with dose modification and supportive care (e.g., preservative-free lubricant eye drops) while patients remained on treatment^11^. These recommendations are also supported by studies with other MMAF-ADCs, which found that treatment-associated corneal changes improved or resolved upon dose modification (reduction and/or delay) or treatment discontinuation^11,37–41^.

Balancing efficacy against the risk of AEs in RRMM management depends on individual considerations for each patient^42^. The RRMM patient care team should be familiar with the potential corneal event risks with belamaf treatment. Referral to an eye care professional is required for regular eye examinations including before treatment initiation and subsequent treatment cycles^23,24^. Depending on the severity of corneal events, management may require belamaf dose reduction and/or delay^23,24^. When possible, it is important for patients to continue treatment in order to maximize survival outcomes. For example, early treatment discontinuation to manage AEs related to immunomodulatory agents may lead to poorer clinical outcomes^42^. Our guidelines have been written to support resumption of treatment following recovery or improvement of corneal events. Going forward, continuing data collection, experience, and insights from hematologist/oncologists and eye care professionals will help to inform these current event management guidelines.

Several avenues of research are ongoing to determine the etiology and potential mitigation strategies of these events. The pathophysiology of the keratopathy (MECs) observed with belamaf and other ADCs is currently unknown^11^. We recently proposed a mechanism whereby keratopathy (MECs) represents an off-target effect of belamaf-induced apoptosis of corneal epithelial cells. Apoptotic corneal epithelial cells are eventually replaced with new epithelial cells, ultimately leading to the resolution of keratopathy (MECs) and symptoms after completion of treatment. This is supported by the apparent migration of the MECs, in some patients, and the evolution of the MECs’ appearance from large clear-spheres to tiny ill-defined flecks. Additional research will help to validate or revise this hypothesis. Furthermore, there is preclinical evidence that belamaf may enter the cornea through the tear film, and exposure-response analyses suggest that belamaf trough concentration may correlate with the probability and timing of corneal events^11,43^. Modeling studies are underway to evaluate the time course of corneal events linked with belamaf pharmacokinetics to determine whether alternative dosing schedules can mitigate corneal exposure. Additional corneal event management strategies are also being investigated.

Knowledge of potential AEs associated with particular therapies may help promote early and effective interventions to prevent and reduce the impact of these events on patients’ quality of life. Close collaboration between hematologist/oncologists and eye care professionals is needed to determine appropriate dose modifications based on the severity of corneal events. These collaborations can also help ensure that patients have easy access to an eye care professional, the eye care professional understands what examinations and assessments are needed, and the hematologist/oncologist receives accurate reports to help appropriately treat their patients. Continued collaboration and effective communication between hematologist/oncologists and eye care professionals are crucial for the effective management of corneal events to ensure that patients receive optimal treatment.

Belamaf is now being investigated to treat RRMM in combination with other anti-tumor treatments, including novel therapies with complementary mechanisms of action. Several of these ongoing studies include a dose exploration phase that will evaluate the belamaf safety and tolerability profile in combination with other anti-tumor treatments. These include the Phase I/II DREAMM-5 platform trial^44^, and the Phase III DREAMM-8 study (NCT04484623)^45^. Other studies such as DREAMM-6 (NCT03544281)^46^, a Phase I/II, open-label, dose escalation and expansion study to evaluate safety, tolerability, and clinical activity of belamaf in combination with approved regimens and DREAMM-4 (NCT03848845)^47^, a Phase I/II, single arm, open-label, two-part study investigating the safety, tolerability and clinical activity of belamaf in combination with a programmed cell death-1 inhibitor pembrolizumab, are also underway. Other ongoing clinical trials include the Phase I/II (NCT03715478), 2-part multi-center, dose escalation study evaluating the maximum tolerated dose, and recommended Phase II dose, safety, tolerability, and efficacy of belamaf in combination with pomalidomide and dexamethasone^48^. These studies should provide further insights into the optimization of belamaf dosing, as well as the effectiveness of mitigation strategies (such as temporary treatment delays), to potentially reduce the frequency, severity, and/or overall impact of corneal events in patients who could benefit from belamaf treatment.

## Data Availability

Information about GlaxoSmithKline’s data sharing commitments and access requests to anonymised individual participant data and associated documents can be requested for further research from ClinicalStudyDataRequest.com.
